# Self-regenerating and hybrid irreversible/reversible PDMS microfluidic devices

**DOI:** 10.1038/srep26032

**Published:** 2016-05-16

**Authors:** Letícia S. Shiroma, Maria H. O. Piazzetta, Gerson F. Duarte-Junior, Wendell K. T. Coltro, Emanuel Carrilho, Angelo L. Gobbi, Renato S. Lima

**Affiliations:** 1 Laboratório de Microfabricação, Laboratório Nacional de Nanotecnologia, Centro Nacional de Pesquisa em Energia e Materiais, Campinas, São Paulo 13083-970, Brasil; 2 Instituto de Química, Universidade Federal de Goiás, Goiânia, Goiás 74001-970, Brasil; 3 Instituto de Química de São Carlos, Universidade de São Paulo, São Carlos, São Paulo 13566-590, Brasil

## Abstract

This paper outlines a straightforward, fast, and low-cost method to fabricate polydimethylsiloxane (PDMS) chips. Termed sandwich bonding (SWB), this method requires only a laboratory oven. Initially, SWB relies on the reversible bonding of a coverslip over PDMS channels. The coverslip is smaller than the substrate, leaving a border around the substrate exposed. Subsequently, a liquid composed of PDMS monomers and a curing agent is poured onto the structure. Finally, the cover is cured. We focused on PDMS/glass chips because of their key advantages in microfluidics. Despite its simplicity, this method created high-performance microfluidic channels. Such structures featured self-regeneration after leakages and hybrid irreversible/reversible behavior. The reversible nature was achieved by removing the cover of PDMS with acetone. Thus, the PDMS substrate and glass coverslip could be detached for reuse. These abilities are essential in the stages of research and development. Additionally, SWB avoids the use of surface oxidation, half-cured PDMS as an adhesive, and surface chemical modification. As a consequence, SWB allows surface modifications before the bonding, a long time for alignment, the enclosure of sub-micron channels, and the prototyping of hybrid devices. Here, the technique was successfully applied to bond PDMS to Au and Al.

Substrates play an important role in microfluidics by affecting the properties and performance of analytical chips because large surface-to-volume ratios are observed in microchannels. Furthermore, the substrates determine the microfabrication strategy. Accordingly, two opposite trends are reflected in microfluidic science and technology: the deployment of high-performance and point-of-use (low-cost, simple, and portable) systems. In general, powerful platforms rely on glass, which requires laborious and expensive microfabrication. Conversely, low-cost microdevices are prepared in a simple way on substrates such as paper, whose performance is lower than glass by focusing on, for instance, applications in capillary electrophoresis, electrochemical detection, organic synthesis, and droplet formation^1^. Here, polydimethylsiloxane (PDMS) is a potential output for deploying powerful and point-of-use platforms, as we will discuss further.

PDMS has greatly contributed to the accessibility of microfluidics in academia and industry by enabling a straightforward and fast process of prototyping. This elastomer is applied as a casting material in replica molding for engraving channels. PDMS is low-cost, optically transparent, and capable of integrating functional units such as valves and pumps^2^,^3^, contributing to the deployment of micro-total analysis systems (μTASs) and lab-on-a-chip (LOC) platforms. Furthermore, such elastomers exhibit outstanding gas permeability, which is important for biological experiments using living organisms or cells^4^,^5^.

Despite the aforementioned advantages, PDMS has some disadvantages such as i) sample contamination with unreacted oligomers, ii) inorganic species absorption, iii) biomolecules adsorption, iv) poor efficiency of separation in microchip capillary electrophoresis (MCE), and v) noticeable swelling in different organic solvents^6^,^7^,^8^,^9^. Nonetheless, these disadvantages can be effectively resolved by adopting practices reported in the literature. Such alternatives include i) bulk modification^10^ or coating of the PDMS surface with SiO_2_^11^ and ii) dissolution in organic media^12^ or extraction^13^ of unreacted oligomers followed by oxidation in air plasma. These approaches generated electroosmotic flows higher than glass. The use of substrates such as thermosets, thermoplastics, paper, and PDMS/glass are additional alternatives^1^. PDMS/glass hybrid chips are largely used because of the compatibility of glass with film integration and functionalization as well as better MCE performance than native PDMS^14^,^15^.

Taking into account the previous discussion, PDMS is a powerful candidate for developing high-performance and point-of-use platforms, especially for the accomplishment of biological tests and the construction of LOC technologies and flexible systems. Meanwhile, we believe a crucial step in the microfabrication poses important challenges to the deployment of powerful and low-cost PDMS chips: the bonding.

PDMS provides a simpler and faster microfabrication process than glass. However, the bonding step exhibits key drawbacks. These methods rely on surface oxidation^16^,^17^, adhesive layer^18^,^19^,^20^,^21^,^22^,^23^,^24^, and chemical modification with diverse groups such as amino/epoxy^25^,^26^, amino/amide^27^, amino/silanol^28^, silanol/silanol^29^ and grafted polymers^30^. Oxidation bonding is carried out primarily in oxygen plasma^16^, but also through corona discharge^17^. Oxidation bonding creates silanol groups by converting –O_2_Si(CH_3_)_2_ to –O_2_Si(OH)_2_. The surfaces undergo an irreversible bonding because of the siloxane covalent bonds (Si–O–Si) that arise from the condensation reactions^31^. Such a method contributed to popularizing microfluidics by providing a simple step for bonding. Conversely, the main disadvantages of this bonding technique are the hydrophobic recovery, harsh plasma conditions, and poor applicability. The oxidative effect in PDMS is temporary owing to the diffusion of oligomers from the bulk to the surface^13^. Hence, the bonding step must be performed within one minute after the oxidation pretreatment^12^. This fact hinders the ultra-large-scale integration (ULSI) and steps of alignment for complex devices^32^. Here, the alternative relies on the removal of PDMS oligomers and the subsequent plasma process^12^,^13^. Such a procedure ensures irreversible bonding as lengthy as 90 h after surface oxidation^12^. However, the harsh plasma conditions damage surface-anchored groups such as receptors for chemical sensors. This phenomenon avoids surface modifications before bonding^33^. The inert and hydrophobic nature of PDMS hinders *in situ* modification processes, especially when patterned and small structures are required^34^. Finally, the oxidation bonding is only applicable to silicon-based substrates. Sealing PDMS onto different substrates can be achieved by surface chemical modification-mediated procedures. These substrates include semiconductors, metals, resists, ceramics, thermoplastics, and alloys^35^,^36^,^37^. Modifications are conducted in the liquid or vapor phase. In general, their protocols are laborious, time consuming, and require oxygen plasma for at least the activation of PDMS. Moreover, unreacted functional groups remain available for the subsequent steps of functionalization, compromising the performance of the device. By contrast, the adhesive bonding approach is fast and low-cost. The glue is based on partially cured (half-cured) PDMS. The substrate is generally pressed against the glue that consists of a PDMS slide or thin film on flat slides^18^,^19^,^20^,^21^. In some cases, the substrate is also half-cured so that both the substrate and flat cover act as glues^22^. A disadvantage is that this process requires time-consuming steps of curing at a low temperature to avoid deformation of the channel. Another strategy is selectively transferring the glue only to the bonding region of the substrate (not to the channel) by a stamping process^23^,^24^. When the adhesive is coated onto an entire substrate, the method’s applicability is limited to PDMS/PDMS microdevices (considering the channels are engraved in PDMS). Alternatively, a selective transferring technique allows this elastomer to bond to other compounds such as glass, semiconductors, metals, and photoresists^32^. Nonetheless, this method provides weak adhesion strengths for PDMS/glass chips, ranging from 0.20 to 0.42 MPa^23^,^32^. Finally, the limitations intrinsic to all of the adhesive bonding approaches are that the dimensions and physical-chemical properties of the channels can be altered by clogging and the presence of glue. Such clogging is created by glue reflow that can be observed even at medium temperatures. In this case, the accomplishment of the process at room temperature is a potential alternative. Conversely, it results in operation times as lengthy as 48 h^32^.

This paper addresses a groundbreaking method for prototyping PDMS microfluidic chips that represents a potential way to fabricate powerful and low-cost PDMS chips. We focused on PDMS/glass because of the diverse advantages of this hybrid material. The developed method bypasses conventional bonding, which relies on irreversible interactions between the substrate (with microchannels) and the cover. Called sandwich bonding (SWB), this method relies on the mechanical confinement of a glass coverslip on a PDMS substrate, utilizing a PDMS/glass/PDMS sandwich assembly as shown in Fig. 1. SWB combines low cost and time consumption with high fabrication performance. Indeed, SWB ensured great adhesion strengths without the use of surface oxidation, half-cured PDMS as an adhesive layer, solvents, or surface chemical modification, requiring only a laboratory oven for enclosing the microfluidic channels. Hence, SWB adds flexibility in surface modifications (before bonding), alignment, enclosing of sub-micron structures, and fabrication of hybrid platforms. SWB is not limited to silicon-based compounds. Its wide applicability was successfully demonstrated through the irreversible enclosing of PDMS channels with gold (Au) and aluminum (Al). In this case, the burst pressures were, to our knowledge, the highest ever recorded. Moreover, lithographic molds ensured rounded and non-contaminated microchannels after repeated use. This rounded profile is an advantage because it improves the actuation of elastomeric valves and favors additional photolithography steps such as thin film deposition and UV exposure inside microchannels^38^,^39^. Finally, SWB is scalable for ULSI manufacturing and the devices astoundingly showed novel properties in the microfluidics field: i) the ability for self-regeneration after successive leakages and ii) hybrid irreversible/reversible behavior, as discussed below.

When the flow rate into microfluidic chips is excessive, the leakage of the fluids is observed and, then, the device becomes unusable. By contrast, after removing the fluid and drying in a laboratory oven, the SWB microchips demonstrated the ability for regeneration. Following successive processes of leakage, the self-regenerated devices were able to withstand satisfactory values of flow rate (pressure). The reversible nature of the SWB chips was achieved after the simple immersion of the device in acetone for removal of the cover of PDMS. Next, it was possible to detach the PDMS substrate and the glass coverslip for reuse. These chips continued to endure high pressures as well. Both the features of self-regeneration and hybrid irreversible/reversible behavior are novel for a microfluidic device. The ability to disassemble is crucial in the research and development stages, especially when the microdevice integrates high-cost functional components, functionalizations need to be repeated, or harsh cleaning processes are necessary^32^,^40^. The main advantage of our platform regarding the reversible bonding techniques reported in the literature relates to the high adhesion strength (~0.70 MPa). Conventional reversible bonding is based on aspiration^41^,^42^, magnetism^43^,^44^, and packing (PDMS is placed between slides of glass)^45^,^46^,^47^. In addition to drawbacks such as additional apparatuses (vacuum pump and magnets) and key restrictions for devices with high densities of microfluidic structures, these methods generate poor adhesion strength, ranging from 0.05 to only 0.16 MPa.

The following assays are included here: mold characterization, channel cross-section imaging, and tests of adhesion strength, self-regeneration, reversibility, and repeatability through measurements of electroosmotic flow. Considerations on the microfabrication steps and theory involved in SWB are outlined next.

## The approach

### Experimental protocol

**Figure 1:**
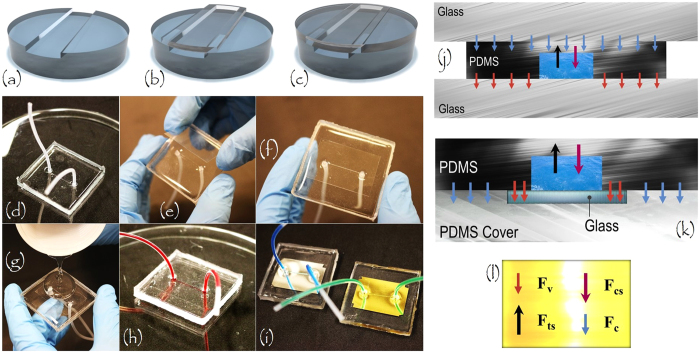
Steps of microfabrication and actuation of forces in devices. PDMS substrate with microchannel obtained by replica molding (**a**,**d**); reversible enclosing of microchannel with glass coverslip (**b**,**e**,**f**); pouring of PDMS cover onto previous structure (**g**); and cure of this cover resulting in a stacked structure of PDMS/glass/PDMS sandwich (**c**,**h**). Substrate integrated silicone tubes for fluid pumping (**d**). SWB was applied for prototyping PDMS/Al and PDMS/Au as well (**i**). Forces in reversible bonded microdevice composed of glass/PDMS/glass sandwich structure (**j**) and SWB PDMS/Glass device (**k**). Discrimination of the force vectors that act over the devices (**l**). Nomenclature: **F**_**v**_, Force related to van der Waals interactions responsible for PDMS/glass self-adhesion; **F**_**ts**_, tensile stress force from liquid; **F**_**cs**_, compressive stress force from PDMS; and **F**_**c**_, counterforce applied by glass cover in (**j**) and PDMS cover in (**k**).

Figure 1(a–i) summarizes the main steps of the SWB process. Initially, a glass coverslip is placed on top of a PDMS substrate, covering the microfluidic channels. This coverslip is smaller than the substrate so that the lateral sides of the PDMS remain exposed. Such a polymer ensures a conformal covering with atomic contact over large areas even in the presence of minor surface perturbations. Subsequently, a liquid composed of PDMS monomers and curing agent is poured onto the stacked structure, covering the exposed sides of the PDMS substrate and the entire surface of the glass coverslip. Penetration of this liquid between the PDMS substrate and the glass coverslip was not observed because of the conformal contact of these two slides. After crosslinking the PDMS cover, the microfabrication is completed. The glass coverslip is confined within a sandwich structure of PDMS/glass/PDMS owing to merging of the PDMS layers (substrate and cover) in the lateral regions of the device. The interface involving these PDMS layers acquires physical chemical properties similar to those of the bulk polymer. This phenomenon is likely due to the crosslinking reactions of unreacted dimethylsiloxane monomers from the substrate with dimethylsiloxane monomers from the uncured cover.

The channels presented a high adhesion strength with burst pressures of up to 0.98 MPa even without irreversible bonding between the substrate and cover. According to the discussion below, this result is likely because of the action of three force components that compensate for the flow-applied tensile strength in the channel. Such forces are related to self-adhesion, compressive stress, and the counter-force from the PDMS cover.

### Theoretical considerations

For glass on PDMS, a reversible bonding is observed through the phenomenon of self-adhesion, which stems from van der Waals interactions. The force related to such interactions (**F**_**v**_) for two slides separated by a distance **D** is given by Hamaker’s model as follows^47^:


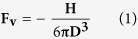


where **H** is the non-retarded Hamaker constant. When a liquid is introduced into the microchannel, it undergoes a tensile stress that contributes to the production of deformations in the PDMS layer. In this case, the tensile stress force (**F**_**ts**_) is balanced by **F**_**v**_ and the compressive stress force (**F**_**cs**_) that represents the dominant adhesive mechanism. Mathematically:


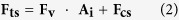


with


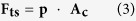


and


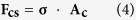


where **p** is the fluid-applied internal pressure, **σ** is the compression stress, **A**_**i**_ represents the area of the slide interface exposed to **p**, and **A**_**c**_ is the area of the microchannel. Substituting (**3**) and (**4**) into (**2**):


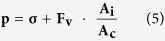


The compression stress is given by:


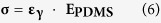


Therefore:


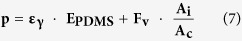


with **ε**_**γ**_ and **E**_**PDMS**_ representing the vertical strain in PDMS and the Young’s modulus of this elastomer (approximately 1.8 MPa), respectively.

For reversibly bonded PDMS/glass assemblies, **p** is not uniformly distributed throughout the PDMS substrate. Otherwise, such a pressure concentrates on the surroundings of the channel to distances smaller than 30 nm (this value determines the calculation of **A**_**i**_). Accordingly, only a reduced fraction of the van der Waals force (**F**_**v**_ · **A**_**i**_) contributes to bonding adhesion. Mathematically, 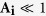, so:


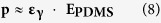


Thus, a low pressure is sufficient to result in large deformations of the elastomer. Such a strain leads to the delamination of the PDMS/glass structure. Indeed, these microchannels withstand only 0.04 MPa according to data obtained from burst pressure tests^38^.

Studies recently reported in the literature showed reversible PDMS/glass channels can withstand pressures of up to 0.16 MPa^47^. For this test, other glass was placed on the PDMS substrate as illustrated in Fig. 1(j), which also displays the forces acting on the device. With PDMS sandwiched between two glass slides, **F**_**ts**_ is balanced by one new component: a counter-force applied by a second piece of glass (**F**_**c**_) that limits the expansion of PDMS. Hence, (**2**) changes to:


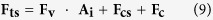


with


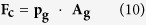


where **p**_**g**_ is the counter-pressure from the second piece of glass and **A**_**g**_ is the area of the PDMS side in contact with this glass slide such that:


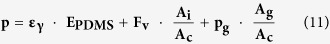


Apart from the production of a new force to balance the fluid-applied internal pressure, the stack composed of glass/PDMS/glass leads to a more uniform distribution of this pressure. Thereafter, an appreciable fraction of **F**_**v**_ begins to assist the adhesive sealing. According to the authors^47^, these phenomena explain the rise in adhesion strength. Mathematically, 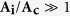 and 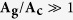, so the contributions from the van der Waals interactions (**F**_**v**_) and the presence of a second glass slide (**p**_**g**_) for balancing the fluid-applied pressure are increased, in agreement with (**11**).

We believe the aforementioned aspects of glass/PDMS/glass structures are valid for microdevices obtained by the SWB process. In this case, a counter-force, **F**_c_, would be applied by the PDMS cover, generating a further increase in adhesion strength by potentializing the contribution of the van der Waals interactions between the PDMS substrate and the glass coverslip. In conclusion, adhesion in SWB is likely related to self-adhesion (reversible bonding), compressive stress from the PDMS substrate, and counter-force from the PDMS cover. The forces acting over the SWB device are illustrated in Fig. 1(k).

## Results and Discussions

### Mold

**Figure 2:**
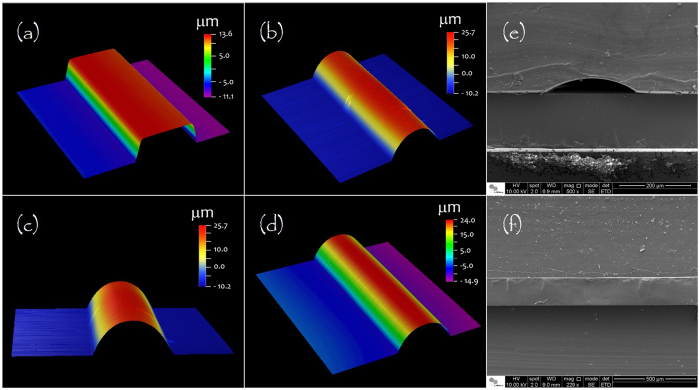
Mold characterization and cross-section images. Profilometry images of positive resist mold before (**a**) and after (**b**–**d**) hardbake. Patterns in AZ 50XT resist were fabricated from metal masks presenting lines with 100 (**a**–**c**) and 500 μm width (**d**). Micrographies show PDMS substrate (in top), glass coverslip (in middle of stack), and PDMS cover (in bottom side) at regions with (**e**) and without (**f**) channel.

Profilometry images of AZ 50XT patterned on glass molds before and after the hard bake are shown in Fig. 2(a–d). This heating process generates a rounded structure with a decrease in width and an increase in height. The rounding of the AZ 50XT photoresist stems from the evaporation of residual solvent. A profile of the pattern is obtained to reduce the surface area^48^.

For metal masks of 100-μm widths, for instance, AZ 50XT patterns were initially 138.3 ± 1.2 μm wide and 21.7 ± 0.3 μm high (*n* = 10). Following the hard bake, these parameters changed to 109.8 ± 0.6 μm and 34.3 ± 0.6 μm (*n* = 10), respectively. For the metal mask of 500-μm width, patterns of the hard-baked molds were 507.9 ± 0.8 μm wide and 33.1 ± 0.3 μm high (*n* = 10). In addition, values of 156.2 ± 0.7 μm and 32.1 ± 0.2 μm (*n* = 10) were achieved for a metal mask presenting a 150-μm width.

As described above, a rounded profile of microchannels represents a positive aspect concerning the deployment of elastomeric valves and the execution of further photolithography steps such as thin film deposition and UV exposure inside the microchannel^38^,^39^.

### Cross-section view

A cross-section of a microchannel fabricated by SWB is shown in Fig. 2(e). This image was recorded by field-emission gun scanning electron microscopy (SEM-FEG). It is possible to verify the dimensions of the channel were not affected by the glass layer, as expected. In addition, problems related to the partial filling of the microfluidic channel by glue, as occurs in adhesive bonding, are not verified here. Thus, pressure and temperature are not critical in the SWB method. Otherwise, these parameters require special attention in adhesive bonding. Although high pressures and temperatures increase the contact surface, eliminating voided areas and, thus, increasing the adhesion strength, such values contribute to a partial filling of the channels by glue owing to the reduction in viscosity of this intermediate adhesive layer^49^. Figure 2(f) portrays a cross-section view of the PDMS/glass/PDMS interface at a region lateral to the channel. Herein, the conformal contact between glass and polymer can be observed.

### Adhesion strength

Leakage tests were conducted to assess the adhesion strength of the SWB microchannels. Aniline-based aqueous dyes were pumped into the microchannels at different flow rates, leading to increasing values of **p**. We used a high performance liquid chromatography pump (HPLC, Shimadzu LC-10AD VP, Columbia, MD) to drive the dyes and measure the pressures attained. Burst pressures (minimum **p** needed to produce delamination of PDMS and, thus, leakages of dye solution) mathematically expressed the parameter of adhesion strength.

**Figure 3:**
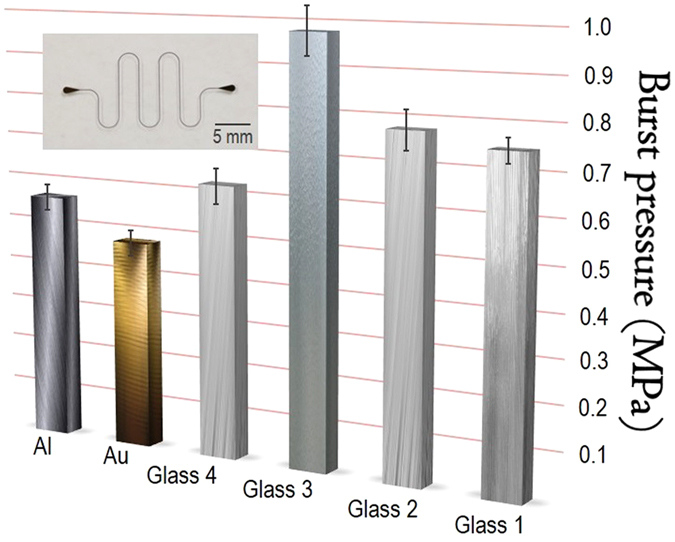
Leakage test for assessing adhesion strength. Burst pressures measured for PDMS/glass (1–4 conditions as highlighted in main text), PDMS/Au, and PDMS/Al SWB chips. Inset: geometry and dimension of channels (100 μm width) employed for test of long-term performance. Lines in top of columns are the error bars (confidence intervals).

The results of the leakage test for evaluating the adhesion strength are shown in Fig. 3. These data were in agreement with our theoretical hypotheses presented above. The improvement in burst pressure for PDMS/glass channels by increasing the merged PDMS area was small. Such a pressure increased from 0.75 ± 0.05 MPa (glass 1; 11.9 cm^2^ area) to 0.78 ± 0.09 MPa (glass 2; 16.1 cm^2^). This result can be explained by considering the two opposite phenomena that affect the adhesion strength. These phenomena are a rise in the counter-force from the PDMS cover (**F**_**c**_) and a decrease in the force from the van der Waals interactions between PDMS and glass (**F**_**v**_) once the area of the merged PDMS increases. Such phenomena contributed, in opposite ways, to the variation in resistance of the channel to the delamination of the PDMS substrate (equation (9)). Conversely, the hard bake appreciably increased the adhesion, producing a burst pressure of 0.98 ± 0.13 MPa (glass 3). We propose that this improvement is due to the increase in **F**_**c**_ from the increased crosslinking density of PDMS in the interface between the substrate and cover. Glass 3’s condition was adopted in all of the following investigations. In the case of the microdevices prepared after storing the PDMS substrate and glass coverslip for four weeks, the burst pressure was reduced to 0.63 ± 0.07 MPa (glass 4). Presumably, this decrease is associated with an increase in the hydrophobic character of the PDMS substrate surface, thus reducing the force from self-adhesion with the glass coverslip.

All of the tested PDMS/glass microchips withstood high pressures compared with other bonding methods reported in the literature. For instance, the maximum adhesion strengths for PDMS/glass were 0.51^50^ and 0.42 MPa^32^ using an oxidative pretreatment by inductively coupled high-density plasma and a partially cured PDMS adhesive, respectively. For functionalization-assisted bonding, the strength was 0.52 MPa with amino/amide functional groups^27^. The best adhesion reported in the literature for PDMS/glass microchannels was attained by utilizing amino/epoxy groups that were coated by chemical vapor deposition^35^. Here, the adhesion strength was higher than 1.03 MPa. Hence, the burst pressure of 0.98 ± 0.13 MPa attained with the hard bake (glass 3) showed that SWB resulted in an outstanding adhesion strength. SWB channels withstood pressures greater than those created by oxidation and adhesive bonding. In addition, such pressures are higher than the highest values observed for most functionalization-based approaches (see Table 1)^25^,^30^.

In SWB, we propose the adhesion level strongly depends on self-adhesion, the compressive stress from the PDMS substrate, and the counter-force applied by the PDMS cover. Thus, it is expected the SWB technique will permit the sealing of PDMS on various non-silicon substrates such as metals, ceramics, and polymers. Such a broad applicability of the deployed method was evaluated by constructing hybrid chips. More specifically, a PDMS substrate was bonded to Au and Al using the glass 3 condition. It is important to highlight that a thin layer of native oxide is rapidly produced on the Al surface when exposed to air^32^. The potential uses of hybrid microchips composed of PDMS include the fabrication of microvalves and heterogeneous devices in the microelectronics and packing fields^37^.

The burst pressures for PDMS/Au and PDMS/Al were measured as 0.48 ± 0.03 MPa and 0.57 ± 0.03 MPa, respectively. This decrease in the burst pressure compared with the PDMS/glass assembly once again shows the relevance of self-adhesion for bonding, in accordance with our previously described hypothesis (equation (11)). Despite this reduction, the burst pressures obtained herein are, to our knowledge, the highest values ever recorded. Functionalization-mediated approaches created maximum pressures of approximately 0.30 (PDMS/Au) and 0.20 MPa (PDMS/Al)^27^,^36^. For adhesive bonding^32^, such pressure values were 0.24 and 0.29 MPa, respectively.

Regarding long-term performance, hard-baked microchips (glass 3) withstood a pressure of 0.80 MPa for 10 h without fracture or leakage. Zigzag microfluidic channels of approximately 150 and 100 μm in width were tested (four chips in each case). These data are relevant in showing that SWB is suitable for prolonged applications.

### Regeneration

**Figure 4:**
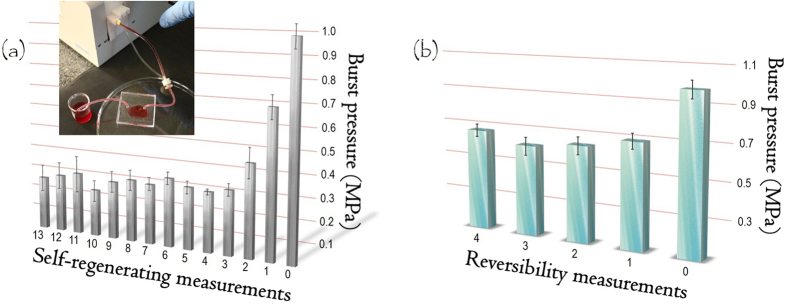
Regeneration and reversibility tests. Burst pressures for PDMS/glass chips after leakages by successively flowing the dye inside channels (**a**) and after successive processes of (i) detachment of PDMS substrate and glass coverslip and (ii) bonding (**b**). Inset: PDMS/glass microdevice with leaked channel after exposure to an excessive flow rate. Lines in top of columns are the error bars (confidence intervals). The first pressure in 0 is relative to the native SWB device.

**Figure 5:**
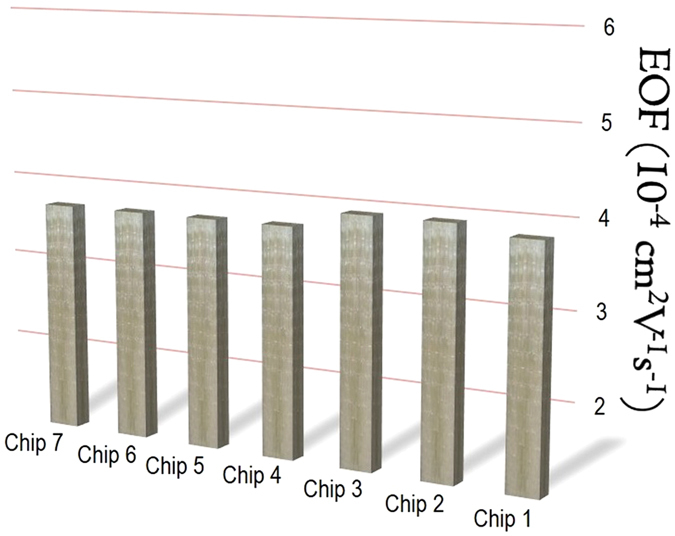
Repeatability test. EOF values determined for SWB PDMS/glass chips. Confidence intervals are not shown because their values for the different microdevices were very low, 9.7 ± 2.5 10^−7^ cm^2^ V^−1^ s^−1^ (*n* = 7).

For pressures higher than the burst pressure, the leakage was confined to the region under the coverslip as illustrated in the inset of Fig. 4(a). This result is due to lateral merging between the substrate and the cover of PDMS. After the removal of the dye solution and drying at 120 °C for 20 min in a laboratory oven, SWB microchips (glass 3 condition) astoundingly demonstrated the ability for regeneration. This property most likely arises from the compressive stress of the PDMS substrate and the counter-force from the PDMS cover. Following the third successive process of leakage, removal of dye solution, and baking, the regenerated devices were able to withstand pressures of 0.28 ± 0.02 MPa according to the burst pressure data shown in Fig. 5 (glass 3). This reduction in adhesion probably occurred as a function of the gradual decrease in the ability of PDMS to uniformly cover the glass. Nevertheless, burst pressures of 0.28 MPa are sufficient to withstand the internal pressures observed in most analyses performed in microfluidics^51^.

To the best of our knowledge, this report on the self-regeneration of microfluidic channels after successive leakages is unrivaled in the literature. Other methods of regeneration are related, in reality, to the reusability of chips following channel-cleaning processes by removing clogging from particles or chemically anchored species. Such methods include anti-fouling coatings^51^, rinsing at high flow rates, harsh chemicals^52^, desorption techniques^53^,^54^,^55^,^56^ and more recently, thermal treatment^57^. The deployment of reversible bonding methods is another powerful output^40^.

### Hybrid irreversible/reversible behavior

In reversible bonding, the slides that compose the channels of the microchip (substrate and cover) can be detached without damaging structures or leaving residues behind^40^. This ability was achieved in SWB by dipping the device (glass 3 condition) into acetone for only 20 min with the aid of ultrasound. Following this process, the PDMS cover was easily removed using tweezers and it was possible to detach the PDMS substrate and the glass coverslip for reuse without any damage or residues. These slides were baked at 95 °C for 10 min in a laboratory oven to remove the acetone and then successively bonded by SWB and detached as illustrated in Fig. 4(b). The devices obtained from the first detachment endured a pressure equal to 0.65 ± 0.03 MPa. This decrease in burst pressure with respect to the native devices (~1.00 MPa) is likely because of the reduction in the density of crosslinking reactions involving unreacted monomers from the same PDMS substrate and monomers from the new PDMS cover that was added after each detachment. Despite this decrease, the obtained adhesion is higher than that observed for conventional reversible bonding^41^,^42^,^43^,^44^,^45^,^46^,^47^. As previously mentioned, these methods produced adhesion strengths of 0.05 up to only 0.16 MPa. More recently, Wasay and Sameoto proposed a potential way to perform reversible sealing^40^. This method relied on conformal contact between a flat slide and a gecko-inspired surface of a polymer. The maximum adhesion strength was 0.66 MPa. This value is very satisfactory, but its fabrication required sequential and relatively laborious steps of photolithography and replica molding.

### Repeatability

The electroosmotic flow (EOF) mobility was determined for seven SWB microchips to assess the repeatability of SWB. The EOF depends on the roughness, dimensions, and composition of the channel inner wall as well as temperature, pH, and the ionic strength of electrolyte solutions^58^. Since the experimental conditions were the same in all of the situations, the obtained data can be attributed solely to the properties of the channels.

Figure 5 shows the results of EOF experiments for PDMS/glass microchips (glass 3 condition), which are in agreement with the literature^59^. Each point in the graph is a global average achieved for four measurements in the seven devices (*n* = 28). There was no appreciable difference among the EOF values that presented an average of 3.8 ± 0.1 10^−4^ cm^2^ V^−1^ s^−1^. Furthermore, the microchannels exhibited stable EOF according to the confidence levels illustrated in Fig. 5 (error bars) for each microchip. Such data indicate a satisfactory repeatability for the SWB method.

Apart from the analysis of the repeatability of the method, the EOF results showed the PDMS channels were not contaminated by resist patterns on the lithographic mold following the repeated use of this mold. As mentioned in the Methods section, four devices (1–4 in Fig. 5) were fabricated by using a new mold, whereas the other three (5–7 in Fig. 5) were produced only after the repeated use for more than 12 h of the same mold. Consequently, AZ 50XT-based molds demonstrated two important advantages compared with SU-8: the creation of rounded features and no contamination of the substrate upon after successive use^9^,^38^. According to Bubendorfer *et al*.^9^, PDMS microchannels fabricated by replica molding on a SU-8 mold are contaminated by hexafluoroantimonic acid, the activated initiator of SU-8 photoresist. This initiator changed the surface zeta potential, leading to variable EOF after a minimum repeated use for 12 h of the same mold.

### Comparative study

Important and generic features of the methods reported in the literature for bonding PDMS to PDMS itself, glass, and non-silicon species are shown in Table 1. These methods rely on surface oxidation, adhesive layer, chemical functionalization, and SWB procedure. SWB stands out above the others insofar as it offers numerous benefits. Furthermore, SWB is the only method that can regenerate and exhibits hybrid irreversible/reversible behavior. Most aspects highlighted in Table 1 were debated in the introduction of this paper. With regard to the operation time, the adhesive method usually provides fast prototyping. Nonetheless, the use of long-term procedures can be required to avoid filling the channels with glue^32^. Additionally, chemical modification is limited to the inner walls of the PDMS microchannel when the glue layer is coated on an entire flat surface. In this case, a broader modification requires the selective transfer of uncured PDMS onto the bonding regions.

We herein report a very simple, fast, low-cost, and groundbreaking method to fabricate PDMS/glass microdevices. Our results represent an important breakthrough concerning the merging of the two trends in microfluidics: the development of high-performance and point-of-use technologies. Our method builds on the pioneering method of plasma-assisted oxidation bonding in enabling the widespread use of microfluidics techniques by providing easy access methods of microfabrication to non-experts.

SWB requires only a laboratory oven for the bonding. Regardless of its simplicity, this method created high-performance microfluidic channels. Such structures featured self-regeneration (after leakages) and hybrid irreversible/reversible behavior, with an adhesion stronger than that obtained by conventional techniques of reversible bonding. These abilities are essential in the stages of research and development. Additionally, the deployed approach avoids the use of surface oxidation, the adhesive of half-cured PDMS, and surface modifications. As a consequence, SWB allows: i) surface chemical modifications before the bonding, ii) a long time for alignment (it bypasses the step for removing PDMS oligomers that can last approximately four days^12^), iii) the enclosure of sub-micron channels, and iv) the prototyping of hybrid devices. Here, the technique was successfully applied to bond PDMS to Au and Al. The burst pressures of these hybrid chips were greater than the values recorded by surface chemical modification-mediated methods. Finally, all of the data of the adhesion test were in accordance with our theoretical hypotheses about SWB.

The questions raised in this paper encourage new investigations, such as more detailed studies about the effect of diverse factors on adhesion strength. These factors include the thickness of stack layers and compounds other than glass, Au, and Al. Additionally, the performance in microchip electrophoresis and the possibility of integrating electrodes for electrochemical detection should be evaluated.

## Methods

### Chemicals

Soda-lime glass slides were purchased from Perfecta (São Paulo, São Paulo, Brazil) whereas isopropanol was acquired from Synth (Diadema, São Paulo, Brazil). Aniline, sodium hydroxide (NaOH), monobasic sodium phosphate (NaH_2_PO_4_), and dibasic disodium phosphate (Na_2_HPO_4_) were supplied by Sigma-Aldrich (St Louis, Missouri, USA). Sulfuric acid (H_2_SO_4_), hydrogen peroxide (H_2_O_2_), AZ^®^ 50XT photoresist, AZ^®^ 400K developer, and hexamethyldisilazane (HMDS) were purchased from Microchemicals (Ulm, Baden-Württemberg, Germany). Sylgard 184 silicone was supplied by Dow Corning (Midland, Michigan, USA). The solutions were prepared in deionized water (Milli-Q, Millipore Corp., Bedford, Massachusetts, USA).

### Mold

Microchannels on PDMS were obtained using replica molding. For this process, the molds were not based on SU-8 as usual but on AZ 50XT positive resist structures patterned onto glass. Briefly, glass was chemically cleaned by immersion in piranha solution (H_2_SO_4_/H_2_O_2_ 2:1, v/v) for 10 min to remove organic impurities. Then, the slide was dehydrated at 120 °C on a hot plate (Tecnal TE-038, São Paulo, São Paulo, Brazil) for 40 min. Subsequently, HMDS-reactive silicone was spin-coated (Headway Research Inc. EC101DT, Garland, Texas, USA) on glass at 2900 rpm for 15 s and 500 rpm for 3 s. A soft bake to remove the solvent was performed at 112 °C for 5 min. Next, an AZ 50XT photoresist was deposited and soft-baked under the same conditions used for the HMDS. The dehydration and soft bake steps improved the glass/resist adhesion by decreasing the surface energy^49^. Subsequently, the slide was exposed to 365 nm UV for 2 min using an exposure dosage of 600 mJ cm^−2^ (Karl Suss America Inc. MJB 3 UV 200, Indianapolis, Indiana, USA) and metal masks (prepared in a laser pattern generator from Heidelberg Instruments μPG 101, Heidelberg, Baden-Württemberg, Germany). Development was then performed after dipping into AZ 400 K developer 1:3 v/v to water under continuous stirring at room temperature for approximately 4 min. Finally, the resist pattern on the glass mold was hard-baked at 140 °C for 10 min. This heating generated rounded profiles as shown by profilometry images (Veeco Dektak 2210, Branson, Missouri, USA).

### Microchannel

The PDMS substrate was prepared by initially mixing elastomer-based monomers with thermochemical crosslinker at a 10:1 m/m ratio. This mixture was degassed under vacuum for 30 min. Subsequently, replica molding-based microchannels were fabricated by pouring uncured elastomer onto the mold with patterned AZ 50XT photoresist. The cure was performed in a laboratory oven (Blue M, Blue Island, Illinois, USA) at 95 °C for 40 min before peeling off the mold. The substrate incorporated microchannels and silicone tubes for the inlet and outlet of fluids. The protocol used for integrating such tubes is detailed in the Supplementary Information. The cured PDMS substrate showed approximately a 2-mm thickness and a 20.3-cm^2^ area.

### Enclosing the microchannel

After carving the channel in PDMS, this polymer was reversibly bonded to a coverslip of glass. Such coverslip was 170 μm thick and smaller than the substrate in area. Thus, an area around the sides of the PDMS elastomer remained exposed, as previously highlighted. Any air pockets between the elastomer and glass were easily removed by applying gentle manual pressure. Subsequently, uncured liquid PDMS was cast on the previous stack (see Fig. 1), flowing to the edges due to the capillary phenomenon. Fluid was not expelled after building up on the slide edges because the surface tension was not exceeded. The purpose of the liquid PDMS cover was to coat the coverslip and lateral sides of the polymer. The thickness of this cover was approximately 800 μm. After planarization of the uncured liquid, the chip was placed in the laboratory oven at 95 °C for 40 min to cure the PDMS cover. The elastomers on the lateral sides merged together, as previously mentioned. Therefore, the interfaces between the PDMS substrate and cover acquired physical chemical properties similar to those of the bulk polymer.

A cross-section image of the enclosed channel was achieved by SEM-FEG. We used FEI Inspect F50 (Hillsboro, Oregon, USA) equipment. The samples were metalized with Au using a BAL-TEC SCD 050 sputterer (Balzers, Oberland, Liechtenstein) to improve image contrast.

### Adhesion strength

In measurements to determine the burst pressures, the microchannels were straight and rounded, presenting approximately a 500-μm width. Additionally, zigzag microchannels (see Fig. 3) approximately 150- and 100-μm in width were used to evaluate the long-term performance of the microdevices. Such chips underwent high internal pressures for 10 h. The dimensions of the lithographic molds were described above.

The adhesion strength of the PDMS/glass SWB devices was investigated by altering the area of the glass coverslips, namely, 8.4 and 4.2 cm^2^. Accordingly, the lateral sides with merged PDMS (substrate and cover) presented areas equal to 11.9 (condition: glass 1) and 16.1 cm^2^ (glass 2), respectively. Such areas presumably affect the adhesion strength by changing the counter-force from the PDMS cover, **F**_**c**_, and the force related to self-adhesion, **F**_**v**_. In addition to the contact area, we also evaluated hard baking the chips after curing the PDMS cover. Here, the merged PDMS had an area of 11.9 cm^2^ (condition: glass 3). The hard bake step was conducted in a laboratory oven at 120 °C for 20 min to complete the crosslinking reactions in PDMS. Finally, the adhesion strength was investigated for chips that were constructed (using the glass 3 condition) after a storage time for the PDMS substrate and glass coverslip of four weeks (condition: glass 4). This test aims to show one more advantage of SWB with respect to the surface oxidation-based method. For this test, the bonding must be conducted within a few minutes after oxidation pretreatment. In each one of the tested conditions (including fabrication with Au and Al, as well as the tests of regeneration, reversibility, and repeatability), the analyses were performed with *n* = 4 on four microchips (globally: *n* = 16 for each measurement in this paper). Confidence intervals were calculated for *α* = 0.05. Glass 3 condition was used in the construction of hybrid chips and tests of regeneration, repeatability, and reversibility. The test of reversibility required the use of an ultrasonic cleaner (Branson Ultrasonics Corporation 1210, Danbury, Connecticut, USA), as discussed above.

### Fabricating PDMS with metals

PDMS substrates with attached channels were enclosed with Au- and Al-coated glass slides to show the wide applicability of the SWB method. The adhesion strength of straight microchannels with approximately 500 μm widths was assessed using the leakage test, as previously mentioned. These metals (100 nm thickness) were attained by coating glass coverslips using an electron beam (Leybold Univex 300, Cologne, North Rhine-Westphalia, Germany). Titanium (20 nm thickness) was used as the adhesion promoter of Au and Al films to the glass surface.

### Repeatability

Once again, the microchannels were straight and rounded with approximately a 500 μm width. A single mold was used in the tests for evaluating the adhesion strength. The intention was to assess the possible contamination of PDMS microchannel walls by AZ 50XT patterns after repeated use of the mold. Of the seven microchips investigated for repeatability assessments, four of them were achieved with new molds. Conversely, the other three chips were fabricated after a repeated use for more than 12 h of the same mold.

Experimentally, PDMS/glass microchannels were initially rinsed with 100 mmol L^−1^ NaOH for 30 min to create negatively charged groups on their walls. Next, cleaning steps with deionized water and phosphate buffer were performed for 20 and 30 min, respectively. The cleaning process was intended to balance the electric double layer and pH on the microchannel walls. Finally, the reservoirs were filled with 100 μL of buffer. Such preconditioning steps were achieved by applying a potential of 50 V. The EOF was determined by the current-monitoring method with phosphate buffer at pH 7^60^. Solutions were prepared using NaH_2_PO_4_ and Na_2_HPO_4_. The measurements relied on alternating runs at two concentrations of buffer (10 and 20 mmol L^−1^) by applying a potential of 100 V. Potentials were applied utilizing a high voltage power supply (eDAQ ER 230, Denistone East, New South Wales, Australia).

## Additional Information

**How to cite this article**: Shiroma, L. S. *et al*. Self-regenerating and hybrid irreversible/reversible PDMS microfluidic devices. *Sci. Rep.* **6**, 26032; doi: 10.1038/srep26032 (2016).

## Supplementary Material

Supplementary Information

Supplementary Information

## Figures and Tables

**Table 1:** Comparative study.

Bonding	Applicability	Cost	Time	Functionalization†	Filling£	ULSIǂ	Storing†	ReuseΩ	Adhesion (MPa)Σ	References
Surface oxidation	Limited€	High	Short	No	No	Yes	No	No	0.40–0.51	16,17,50
Adhesive	Moderate	Low	Moderate	Moderate	Yes	Moderate	Yes	No	0.20–0.51	18, 19, 20, 21, 22, 23, 24,31
Functionalization	Wide	High	Long	No	No	No	Yes	No	0.18–1.03	25, 26, 27, 28, 29, 30,34, 35, 36
SWB	Wide	Low	Short	Yes	No	Yes	Yes	Yes	0.63–0.98	This paper

Comparison of some parameters related to methods for bonding PDMS to PDMS itself, glass, and non-silicon compounds.

^€^Limited to silicon-based compounds.

^†^Before bonding.

^£^Alteration in dimensions of microchannel.

^ǂ^Viability for ULSI processes.

^Ω^Regeneration after successive leakages and reversible property.

^Σ^Burst pressure.
