# Supplementary material for: Self-regenerating and hybrid irreversible/reversible PDMS microfluidic devices
Source: Sci Rep. 2016 May 16;6:26032. doi: 10.1038/srep26032 (PMC4867595; doi:10.1038/srep26032)
Supplement: Supplementary Information [file srep26032-s1.doc]

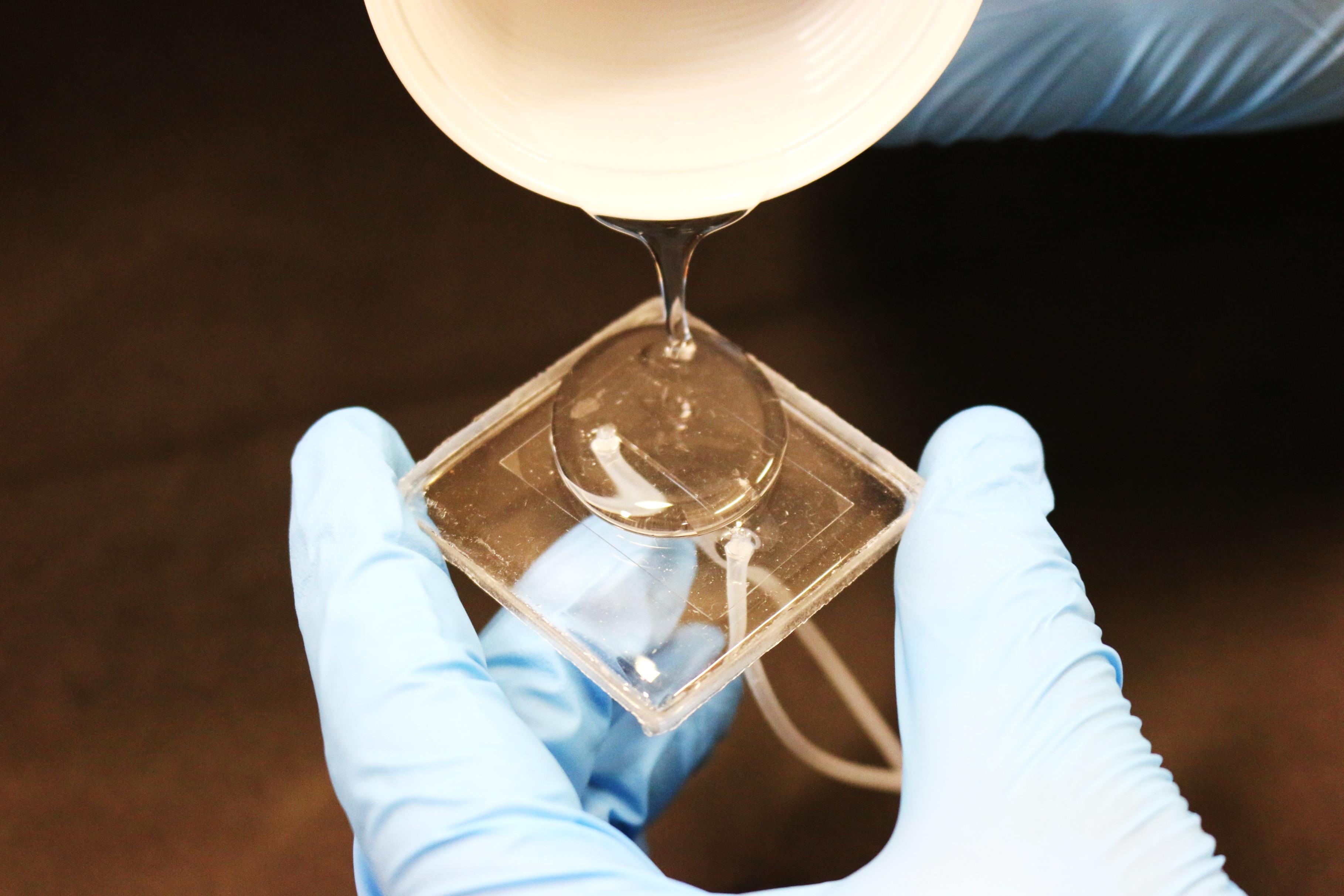


Supplementary Information

**Self-regenerating and hybrid irreversible/reversible PDMS microfluidic devices**

Letícia S. Shiroma,^a^ Maria H. O. Piazzetta,^a^ Gerson F. Duarte-Junior,^b^ Wendell K. T. Coltro,^b^ Emanuel Carrilho,^c^ Angelo L. Gobbi,^a^ and Renato S. Lima*^a^

^a^ Laboratório de Microfabricação, Centro Nacional de Pesquisa em Energia e Materiais, Campinas, São Paulo, 13083-970, Brasil. E-mail: **renato.lima@lnnano.cnpem.br**.

^b^ Instituto de Química, Universidade Federal de Goiás, Goiânia, Goiás, 74001-970, Brasil.

^c^ Instituto de Química de São Carlos, Universidade de São Paulo, São Carlos, São Paulo, 13566-590, Brasil.

Integration of tubes in PDMS substrate

Main steps of replication process in PDMS by soft lithography are shown in **Fig. S1**. At first, PDMS monomers (**1**) were mixed to its cure agent (**2**) at 10:1 weight ratio **(A)**. During the homogenization of this mixture by mechanical stirring, there is the formation of bubbles owing to high viscosity of monomers which reduces the diffusion through the liquid. To remove such bubbles, the bottle (a plastic cup in this case) with the mixture was degassed in *glass desiccator (****3****,****4****)* *connected* *to a* *vacuum pump* for 30 min **(B)***. Afterwards, the mixture was dumped on mold* ***(C)****. Herein, a metal structure was employed to delimit dispersion of uncured PDMS that* flows because capillary phenomenon*. In addition, silicon tubes (1.0 mm d.i.) were aligned in relation to patterns on mold with the aid of holes in acrylic pieces (****5****). These tubes were intended to access the reservoirs of microchip* for inlet and outlet of fluids*. To avoid the filling of tubes by the liquid of monomers, nails were inserted in down sides of tubes. Continuing, the* cure of PDMS (**6**) was realized at 95 °C for 40 min on a greenhouse (it was used in this paper rather than hot plate) ***(D)***. PDMS was, then, easily demolded ***(E)***. For this, the polymer edges were initially detached from metal structure by using a scalpel (**7**) for subsequent process of demolding (**8**,**9**). Finally, the nails were removed from silicone tubes **(F)** through tweezer (**10**) and fingers (**11**,**12**).

**
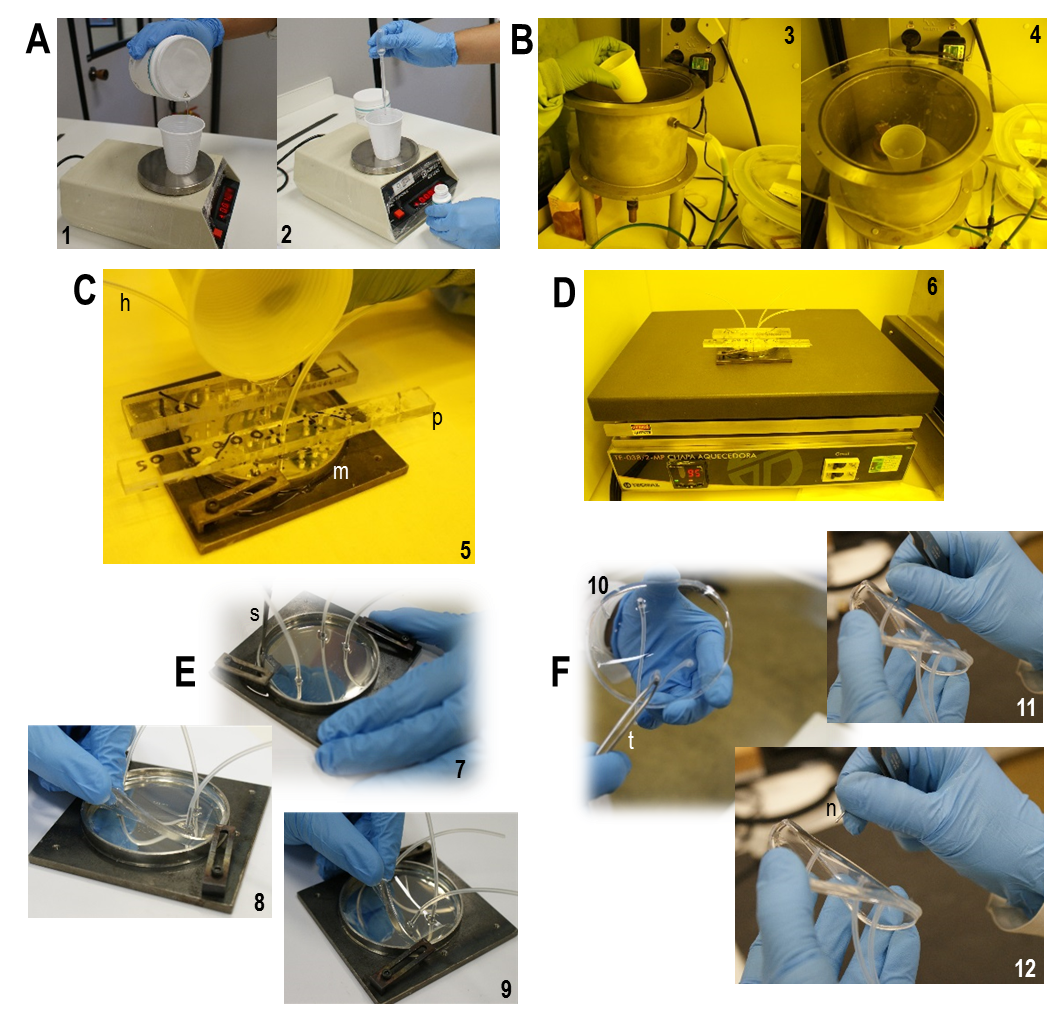
**

**Fig. S1.** Photos showing the main replication steps of the soft lithography process to carve microchannels in PDMS. In this illustrative case, the microchannels were Y-shaped containing approximately 100 μm width and 50 mm depth. Definitions: **m**, metal structure to delimit dispersion of uncured PDMS; **h**, tubes for inlet and outlet of fluids; **p**, piece of acrylic with holes to align the tubes; **s**, scalpel; **n**, nail; and **t**, tweezer.

**Video: “SWB.avi”**

Main steps of SWB microfabrication and obtained microdevices.
